# Distinct Predictors of Social Visitation vs Care Coordination Visitation of LTC Facility Residents

**DOI:** 10.1093/geroni/igaf122.2166

**Published:** 2025-12-31

**Authors:** Caroline Collins-Pisano, Rachel Weiskittle

**Affiliations:** University of Colorado Colorado Springs, Colorado Springs, Colorado, United States; University of Colorado, Colorado Springs, Colorado Springs, Colorado, United States

## Abstract

Loneliness is pervasive among older adults residing in long-term care (LTC) facilities despite frequent familial visitation. Extant research on LTC visitation assess social visitation (e.g., talking with the resident about their day) and care coordination (e.g., provision of personalized care) as one construct. It is possible these two forms of interaction impact resident loneliness differently. Past studies have identified barriers to overall visitation. The purpose of the current study was to investigate if predictors of overall LTC visitation were associated with social visitation distinct from care coordination. 175 community-dwelling adults aged 18 and older with a close friend or relative residing in a LTC facility were recruited via ResearchMatch to complete an online survey regarding demographics, LTC visiting behaviors, and barriers of engagement with LTC residents. Multiple regressions were conducted to assess the relationships between known predictors of overall LTC visitation with social visitation and care coordination. Our collective findings support our hypothesis that social visitation and care coordination are distinct types of LTC visitation. LTC facility characteristics and residents’ communication and functional abilities predicted social visitation but not care coordination. Characteristics of the participant and resident relationships predicted both social visitation and care coordination. Demographic variables and participants’ perceptions of aging, LTC facilities, and the resident’s quality of life predicted neither social visitation nor care coordination. This paper presentation will also discuss the clinical implications of these preliminary findings, such that programs targeting resident loneliness may warrant tailoring towards specific predictors of social visitation over care coordination.

